# Assessing Pharmacy Students’ and Preceptors’ Understanding of and Exposure to Antimicrobial Stewardship Practices on Introductory Pharmacy Practice Experiences

**DOI:** 10.3390/pharmacy8030149

**Published:** 2020-08-20

**Authors:** Sara Revolinski, Jacqueline Pawlak, Ciara Beckers

**Affiliations:** 1 School of Pharmacy, Medical College of Wisconsin, Milwaukee, WI 53226, USA; jpawlak@mcw.edu (J.P.); cbeckers@mcw.edu (C.B.); 2 Department of Pharmacy, Froedtert & the Medical College of Wisconsin–Froedtert Hospital, Milwaukee, WI 53226, USA

**Keywords:** antimicrobial stewardship, pharmacy, pharmacy student, education, survey, experiential education

## Abstract

Antimicrobial stewardship (AMS) is commonly employed, and may be required, in multiple healthcare settings, with pharmacists playing an integral role in developing and conducting AMS techniques. Despite its prevalence, AMS is minimally taught in pharmacy school curricula. In order to increase student and preceptor understanding and application of AMS techniques, the Medical College of Wisconsin School of Pharmacy required introductory pharmacy practice students to complete three checklists and reflections of AMS techniques observed at three different practice settings: inpatient, ambulatory, and community (retail) pharmacy. Student and preceptor understanding and application of AMS techniques were then assessed via voluntary survey. Survey response rates were 43% for pharmacy students, while preceptor response rates were 27%. Student understanding and application of AMS techniques increased after completion of the AMS checklist, with the largest magnitude of change seen with antibiotic selection recommendations and guideline and policy development. Preceptor understanding was minimally impacted by the activity; however, an increase in understanding was seen for allergy assessments, antibiotic time-outs, and vaccine assessments and recommendations. AMS is an important component of pharmacy practice today. Implementation of a checklist and reflection activity within experiential education increases perceived student understanding and application of relevant AMS techniques.

## 1. Introduction

Antimicrobial stewardship (AMS) is commonly seen in many facets of healthcare, and pharmacists are important members of an antimicrobial stewardship team [1]. Since 1 January 2017, The Joint Commission (TJC) has required all acute care facilities to comply with the antimicrobial stewardship medication management standard [1]. Additionally, on 1 January 2020, TJC also implemented AMS requirements for accredited ambulatory care centers [2]. Despite these widespread requirements for pharmacy practice, no consensus exists for AMS education in pharmacy education. The American College of Clinical Pharmacy published a curriculum toolkit in 2016 identifying AMS as one of 24 Tier-2 infectious disease competencies, recommending students receive education and training on AMS but suggesting additional postgraduate training may be required [3]. AMS has since been removed from the 2019 version of the toolkit [4].

Several frameworks for incorporating AMS into pharmacy education have been proposed. Gallagher and colleagues recommend incorporating AMS concepts and terminology throughout the didactic instruction via pharmacology, microbiology, therapeutics, and social and administrative courses [5]. Case-based learning should be utilized to demonstrate AMS concepts. In addition to didactic education, Chahine et al. proposed that AMS education should be deliberately incorporated into experiential education for pharmacy learners [6]. AMS education, dose optimization, and intravenous (IV) to oral (PO) interchange were recommended for both introductory pharmacy practice experience (IPPE) students and advanced pharmacy practice experience (APPE) students. APPE students were additionally recommended to participate in guideline and order set developments, assessing combination therapy, and de-escalation. Of note, prospective audit and feedback and antimicrobial restrictions were recommended strategies for postgraduate pharmacy learners.

In 2018, a survey was sent to infectious diseases faculty or department chairs at 137 schools of pharmacy to assess incorporation of AMS within the curriculum [7]. A total of 116 schools participated. AMS was incorporated into the required didactic curricula for 68.1% of the respondents, and the elective didactic curricula for 37.1% of respondents. Lectures and case-based instruction were the most common pedagogies utilized in both required (93.7% and 57.0%, respectively) and elective (86.0% and 83.7%) didactic courses. AMS was incorporated into the experiential curricula for 83.6% of respondents, primarily because an elective experiential rotation was offered. Respondents noted the most common activities performed by students on experiential rotations included de-escalation (96.9%), dose optimization (95.9%), duration of therapy optimization (90.7%), prospective audit and feedback and/or antimicrobial restriction (88.7%), and IV to PO interchange (85.6%). AMS education within schools of pharmacy was more commonly reported in schools that employed a faculty member who specializes in AMS compared to those that did not (88.1% vs. 71.9%, *p* = 0.049). This survey did not assess the impact of the didactic or experiential education on student learning.

Castro-Sanchez and colleagues assessed incorporation of AMS education in pharmacy school programs in the United Kingdom [8]. Pharmacy schools were most likely to incorporate instruction related to minimizing unnecessary antibiotic use, timing of antibiotic administration, therapeutic drug monitoring, use of IV antibiotics, and microbiologic techniques. The most common pedagogies included didactic lecture with the use of case studies, while some schools also utilized the clinical setting, however the activities utilized in the clinical setting were not further defined.

Justo and colleagues investigated pharmacy students’ knowledge and attitudes about antibiotic appropriateness via a survey [9]. A total of 579 pharmacy students from 12 different pharmacy schools participated. This study also assessed student perceptions on how well pharmacy education prepared them for conducting AMS techniques. Fifty-four percent of students felt their education was good or very good for preparing them to de-escalate antimicrobial therapy, 52% felt their education was good or very good for interpreting antibiograms, and 51% felt their education was good or very good for switching from IV to PO therapy. Only 26% of respondents felt their education was good or very good for working with a patient who demands antibiotics when antibiotic therapy is unnecessary.

As no standard for AMS education in pharmacy education exists, schools of pharmacy have demonstrated varied approaches. These approaches largely include required and elective didactic instruction and elective experiential education rotations. The aim of this study is to determine the impact of a required AMS checklist and reflection activity, embedded within the introductory experiential education curriculum, on student and preceptor understanding and practice of AMS techniques. In addition, this activity will allow students and preceptors to assess the current state of AMS practices at clinical sites and to evaluate which techniques could further be implemented.

## 2. Materials and Methods

The Medical College of Wisconsin (MCW) School of Pharmacy features an accelerated, three-year curriculum, with the first two years dedicated primarily to didactic instruction and the final year dedicated to clinical practice, namely the advanced pharmacy practice experiences (APPEs). In addition to didactic instruction, introductory pharmacy practice experiences (IPPEs) are intentionally woven into the first two years. Students complete a total of seven IPPE experiences; each IPPE experience is 10 weeks in duration with the student attending the practice site every Friday for a minimum of 8 h per day. Students are required to complete two community pharmacy rotations (one at a retail chain location and the other at a non-chain location), two hospital pharmacy rotations (one at Froedtert Hospital, an academic medical center, and the other at a community hospital), two elective rotations, and one interprofessional rotation. Elective rotations may include, but are not limited to, ambulatory care centers, specialty pharmacy, long-term care pharmacy, and inpatient specialty practice such as infectious diseases or oncology. Interprofessional rotations primarily occur in ambulatory or inpatient practice settings, and students are precepted by a non-pharmacist healthcare professional during this rotation.

Didactic courses are delivered in an integrated fashion, with pharmacology, medicinal chemistry, and therapeutics enveloped into the same course. Pharmacy students complete two, 10-week, 5-credit courses dedicated to infectious diseases, which occur during the second semester of their first academic year. AMS is formally taught during a 2-h session within the first infectious diseases course, with AMS topics then integrated throughout the remainder of the infectious diseases curriculum. In preparation for the formal 2-h AMS didactic instruction, students are required to read the executive summary of the Infectious Diseases Society of America’s Guidelines for Implementing an Antimicrobial Stewardship Program [10]. In the classroom, students receive a short presentation outlining the rationale for AMS and are then broken into groups and assigned an AMS technique to research in detail. The groups then develop slides via a template provided that describe their assigned strategy, and finally present their strategy to the class at large. An AMS pharmacist faculty member is present to add or clarify necessary information.

In order to connect this didactic learning with clinical practice, students are also assigned three AMS activities during their IPPE rotations. Students must assess AMS strategies utilized at three different practice settings: inpatient, ambulatory clinic, and community (retail) pharmacy. All students utilize standardized checklists provided by the Centers for Disease Control and Prevention (CDC) for the inpatient [11] and ambulatory rotations [12], and a faculty-developed survey for the community rotation, with components that align with the standardized CDC surveys but that are modified for community/retail practice, as a standardized CDC checklist for the community setting does not exist (Appendix A). After completing each checklist, the student must also compose a 500-word reflection that describes what additional AMS strategy could be implemented by that clinical practice site. This activity is assessed by MCW faculty and not by preceptors. Students were encouraged to discuss this activity with preceptors during completion, but preceptor oversight and involvement is not required. Additionally, preceptors are able to view the activity submission within the learning management software utilized for experiential education student evaluation.

In February 2020, a survey housed within Qualtrics was sent via email to all current MCW School of Pharmacy students in the classes of 2020, 2021, and 2022, in order to analyze the impact of the AMS IPPE educational activity as a means of curricular analysis and development. Additionally, Qualtrics was used to administer a survey to all clinical preceptors who had precepted an IPPE student in the same three classes, to evaluate the indirect impact on preceptors by students completing this activity at their site. Survey questions evaluated the impact of assessing AMS techniques currently employed in clinical practice sites via a checklist paired with a reflection activity on student and preceptor understanding of AMS techniques. Additionally, students and preceptors assessed opportunities for AMS practice expansion at clinical sites. The surveys were designed by experiential education and AMS faculty members and were reviewed by the MCW School of Pharmacy Research Committee and Institutional Review Board. The student and preceptor surveys can be found in Appendix B.

Survey responses were anonymous and de-identified data were analyzed. Descriptive statistics will be used to describe data. Student t-test will be used for continuous, normally distributed data. A *p*-value < 0.05 will be considered statistically significant.

## 3. Results

### 3.1. Pharmacy Student Survey

A total of 60 of 139 eligible students completed the AMS student survey, resulting in a 43% completion rate. Twenty-eight percent of respondents were in the class of 2020, 43% in the class of 2021, and 28% in the class of 2022. Hospital checklists and reflections were completed the most frequently, with 42% of students reporting completion; 21% of students completed the activity in the ambulatory environment, and 37% of students completed the activity in the community setting.

When asked the likelihood of implementing AMS techniques in their future career, only one student thought doing so would be highly unlikely. Fourteen students (33%) felt they were highly likely to implement AMS techniques during their career, 21 students (50%) felt they were likely to implement, and 6 (14%) were unsure.

**Figure 1:**
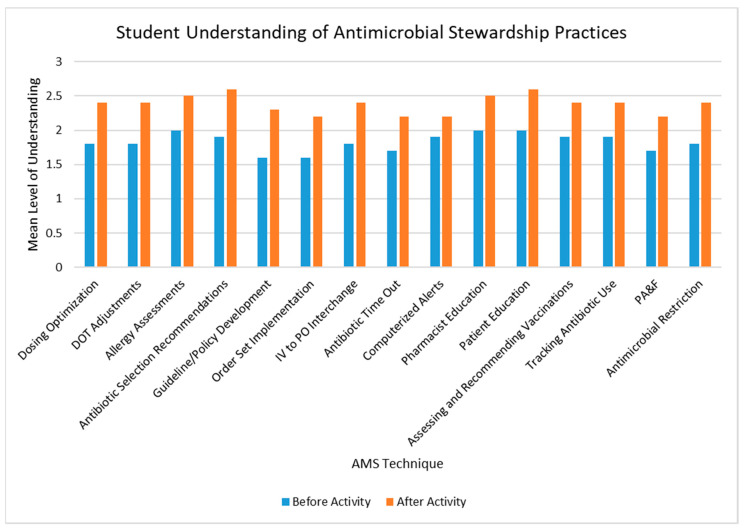
Progression of student understanding of antimicrobial stewardship techniques after implementation of the introductory pharmacy practice experience AMS activity. Understanding was rated on a scale of 1 to 3, with 1 indicating the student “does not understand the AMS technique,” 2 indicating the student “can explain the AMS technique,” and 3 indicating the student “can explain and conduct the AMS technique in practice.” Student report of understanding was statistically significantly increased after the AMS activity (all *p*-values < 0.05). DOT: duration of therapy; IV: intravenous; PO: oral; PA & F: prospective audit and feedback; AMS: antimicrobial stewardship.

Students’ understanding of all AMS techniques increased after completion of the AMS checklist, with the largest magnitude of change seen with antibiotic selection recommendations and guideline and policy development (see Figure 1). In addition, after completion of the reflection component of the activity, 4 (10%) and 26 (65%) students reported that their understanding of AMS practices was greatly enhanced and somewhat enhanced, respectively.

**Figure 2:**
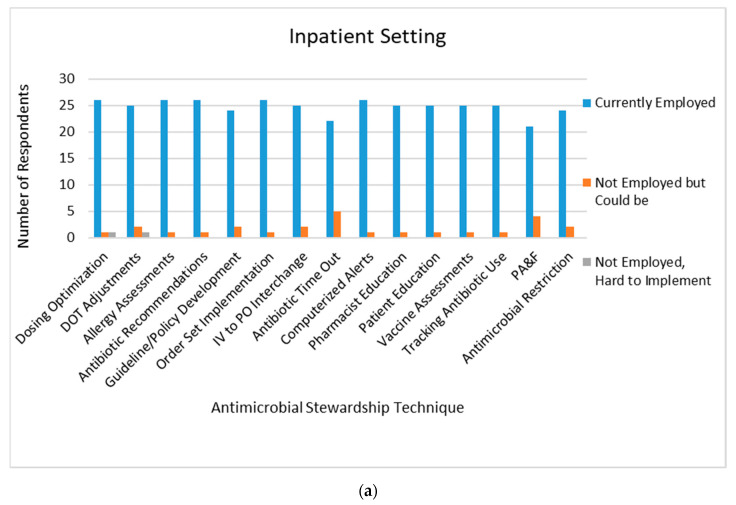

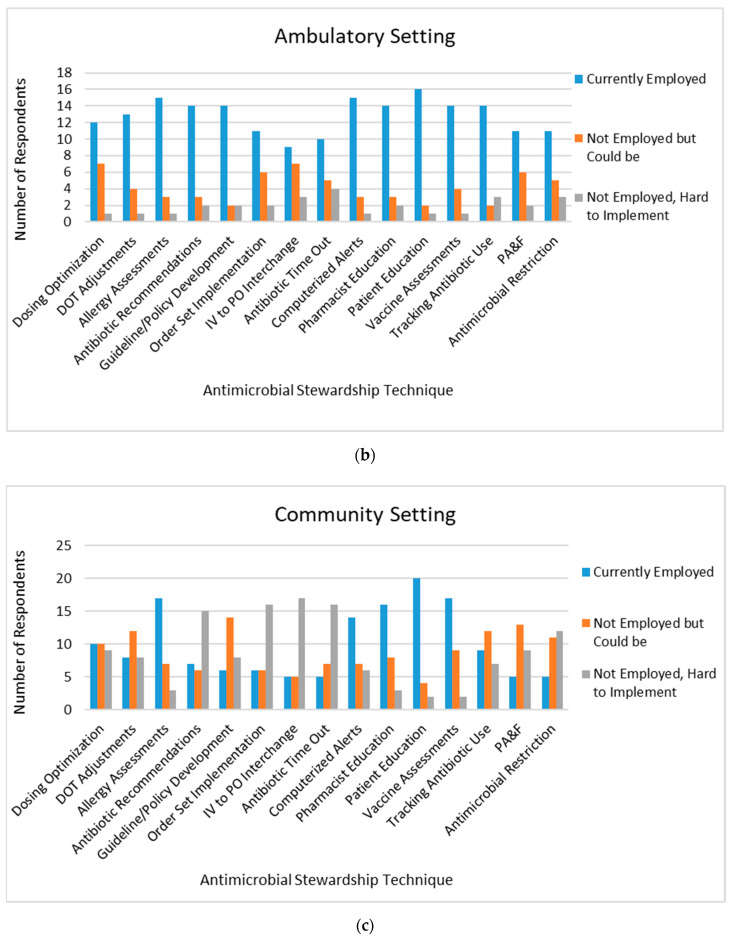
Antimicrobial stewardship techniques observed as currently employed, not employed but could be implemented, and not employed but would be hard to implement at (**a**) inpatient pharmacy sites, (**b**) ambulatory clinic sites, and (**c**) community pharmacy sites, as described by pharmacy students. DOT: duration of therapy; IV: intravenous; PO: oral; PA&F: prospective audit and feedback; AMS: antimicrobial stewardship.

When specifically analyzing data from students on an inpatient rotation, 70.6% of respondents reported observing AMS practices daily. The majority of AMS techniques were already implemented at inpatient sites, however students responded antibiotic time-outs and prospective audit and feedback could be easily implemented at sites where these practices were not in place (Figure 2a).

AMS techniques were not as prevalent in an ambulatory care setting, with 39% of students observing them daily, 39% of students observing them once per month, 17% observing once per rotation, and one student never observing AMS techniques in practice. While ambulatory sites did demonstrate implementation of multiple AMS techniques, students felt sites could also implement dosing optimization strategies, order sets, IV to PO interchange, and prospective audit and feedback (Figure 2b).

Routine observation of AMS techniques in a community setting was varied, with 31% of students observing them daily, 27% observing them once per month, 31% observing once per rotation, and 12% never observing them. Patient education and allergy assessments were the most common techniques used in this setting (Figure 2c). Students identified several techniques that could be implemented in community practice, including dosing optimization, duration of therapy assessments, guideline and policy development, and tracking of antibiotic use. Students also identified that some techniques would be difficult to implement in this setting, namely antibiotic time-outs, antibiotic selection recommendations, and antimicrobial restrictions.

In all, 60%, 33%, and 51% of students reported discussing this activity with their preceptor on the inpatient, ambulatory, and community IPPE rotations, respectively.

### 3.2. Preceptor Survey

Overall, 63 of 236 pharmacist preceptors completed the AMS survey, demonstrating a 27% completion rate. The majority of respondents were inpatient pharmacists, followed by community pharmacists and ambulatory pharmacists at 46%, 40%, and 8%, respectively, which mirrors the practice sites where students reported completing the AMS activities. Forty percent of respondents reported receiving didactic instruction on antimicrobial stewardship in pharmacy school. While only 28% of respondents discussed this IPPE activity with their students, 52% were interested in working on this activity with their IPPE student.

**Figure 3:**
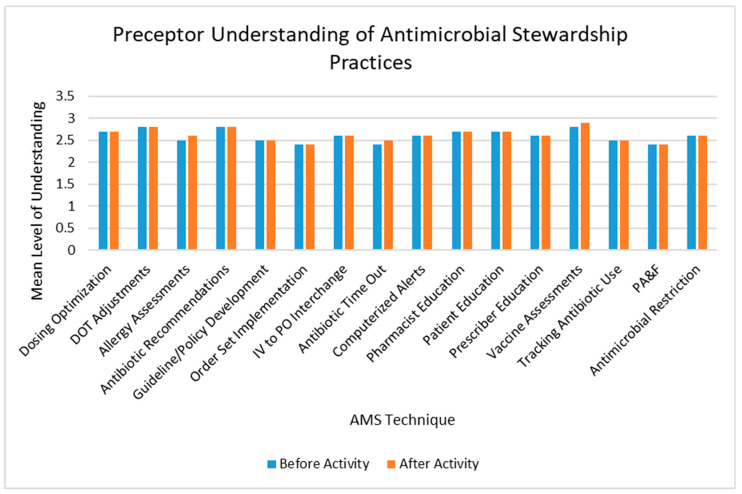
Impact of the antimicrobial stewardship introductory pharmacy practice experience activity on preceptor understanding of AMS techniques. Understanding was rated on a scale of 1–3, with 1 indicating the student does not understand the AMS technique, 2 indicating the student can explain the AMS technique, and 3 indicating the student can explain and conduct the AMS technique in practice. Preceptor understanding did not significantly change after implementing the AMS activity (all *p*-values > 0.05). DOT: duration of therapy; IV: intravenous; PO: oral; PA&F: prospective audit and feedback; AMS: antimicrobial stewardship.

The IPPE AMS activity had minimal impact on pharmacists’ understanding of antimicrobial stewardship practices (Figure 3). Of the 16 practices assessed, only three demonstrated increased understanding after the activity, including allergy assessments, antibiotic time-outs, and assessing and recommending vaccinations.

**Figure 4:**
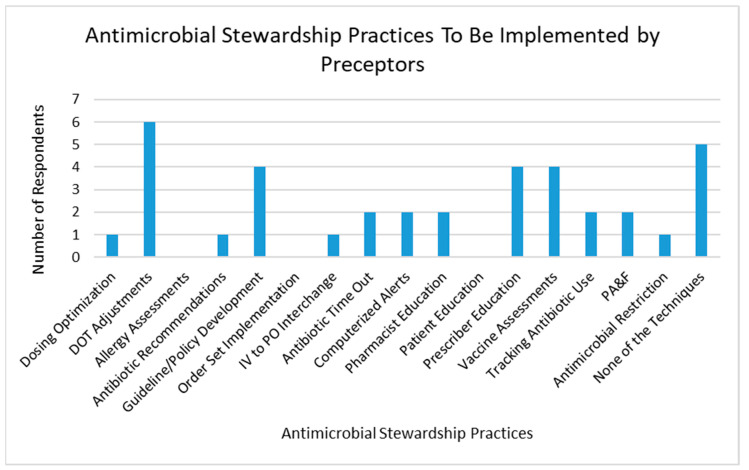
Antimicrobial stewardship techniques pharmacist preceptors would be most likely to implement at their practice site. DOT: duration of therapy; IV: intravenous; PO: oral; PA&F: prospective audit and feedback.

When asked which AMS technique preceptors they were likely to implement that was not currently employed, the most common responses were duration of therapy adjustments, guideline and policy development, prescriber education, and assessing and recommending vaccinations (Figure 4).

## 4. Discussion

Our results demonstrate a required AMS checklist and reflection activity during IPPE rotations significantly increases perceived student understanding of AMS techniques. Students had the greatest exposure to AMS during an inpatient rotation, where these practices were commonly integrated into daily practice. Preceptor survey results indicate a slight trend toward increased understanding of select techniques; however, knowledge was largely unchanged by the activity. The benefit of this activity to preceptors may be underestimated, as not all students discussed this activity with their preceptors. Moving forward, preceptors will be trained on this activity and their involvement will be encouraged, which may help to promote the implementation of AMS techniques at clinical practice sites.

Previous studies have evaluated various instructional methods for AMS in schools of pharmacy; however, none of them have evaluated a required checklist and reflection activity incorporated into IPPE rotation experiences. The University of California, San Francisco, implemented a required didactic AMS learning activity incorporating an online educational module and interprofessional workshop with second-year medical students and third-year pharmacy students [13]. The educational module required students to individually review a branching-logic case and answer associated questions. Following the individual component, students were divided into small interprofessional groups to re-work the first case previously provided online plus an additional case; a large group discussion followed. Students’ knowledge of and attitudes toward AMS were assessed via survey before and after the educational activity. A total of 84.5% and 92.7% of students, respectively, agreed or strongly agreed that the online module and workshop were valuable learning experiences. Survey results showed the curriculum significantly prepared them to describe the role of various professions in appropriate antibiotic use, communicate and engage with the interprofessional team, and to describe collaborative approaches to antibiotic use. This study demonstrated that students developed skills necessary for conducting AMS techniques but did not specifically assess students’ understanding of various AMS techniques as our study did.

While studies have not assessed the incorporation of IPPE students into AMS practice on experiential rotations, APPE students have been utilized to conduct prospective audit and feedback during elective experiential rotations. Benson described his experience incorporating APPE students into prospective audit and feedback activities at a long-term-care hospital [14]. Under the supervision of an infectious diseases pharmacist, APPE students reviewed patient health records to identify opportunities for antimicrobial dose optimization, appropriate durations of therapy, and antibiotic use in the setting of allergies. APPE students also monitored patient response to antibiotic therapy and evaluated microbiologic data to ensure antibiotic use was optimized. Interventions were discussed with the infectious diseases pharmacist preceptor and then presented to the prescriber by the APPE student. While an analysis of student learning was not described in this study, reduced antimicrobial costs per patient day were seen after incorporation of APPE students ($75.37 ± $11.85 prior to implementation, and $64.13 ± $13.27 after implementation, *p* = 0.022). Laibel and colleagues described the integration of APPE students into prospective audit and feedback activities on the medical/surgical floor of an acute care hospital [15]. Students assessed antimicrobial therapy and discussed recommendations with an infectious diseases physician three times per week. The infectious diseases physician then presented those recommendations to the primary team. Over two years, a total of 554 recommendations were made with a 68.4% acceptance rate. The majority of interventions resulted in antimicrobial agent changes or discontinuation. Neither study assessed student understanding of AMS techniques at large or assessment of antimicrobial stewardship activities seen in practice, as our study did. Additionally, these experiential education experiences were only available to students who elected to complete these particular rotations and not to the entire pharmacy class. While our checklist and reflection activity did not actively engage students in practicing AMS techniques, it provided a valuable framework to all introductory pharmacy students by expanding understanding and evaluation of AMS techniques within various practice sites. This framework will assist students when they progress to APPE rotations and are actively involved in practicing AMS strategies. Additionally, our activity exposed students to AMS practices in a variety of practice settings, while literature has primarily described student AMS experiential learning in an acute care environment.

There are several limitations to our study. First, this study is based on survey responses and is dependent on the opinions and perceptions of those that completed the survey. Students and preceptors who already have an interest in or commitment to antimicrobial stewardship may have been more likely to complete the survey, and our relatively low response rate could have resulted in selection bias. There also may have been a tendency for students to overestimate their perceived understanding, as they presume understanding should increase after an educational activity; this phenomenon was not seen in the preceptor responses. Since the majority of preceptor respondents practiced within an acute care environment, they likely conduct AMS techniques in daily practice and this activity likely did not change their understanding. Additionally, the survey was not validated, it was simply created for curricular analysis and development. Our IPPE students typically complete rotations within 60 miles of the school, and the survey results may be influenced by practice in the greater Milwaukee area. All students complete one hospital IPPE rotation at Froedtert Hospital, the academic medical center in Milwaukee, and survey results could be skewed to reflect student experiences at that site, as the majority of students reported completing the acute care activity within the survey. Froedtert Hospital has a robust AMS program, which allowed students to observe most of these techniques in practice.

## 5. Conclusions

AMS, while an integral component of clinical practice today, is inconsistently taught during pharmacy school, with some schools incorporating AMS into the required didactic curriculum and others incorporating it into the elective experiential education curriculum [7,8,13,14,15]. National guidance for how to incorporate AMS education into schools of pharmacy does not exist [4], and schools incorporating this education typically have a faculty member who practices in infectious diseases [7]. This study demonstrates didactic AMS instruction coupled with a required application activity during IPPE rotations can increase perceived understanding and application of AMS techniques by pharmacy students in inpatient, ambulatory, and community practice. This foundational understanding provides a strong framework to build upon when conducting AMS in clinical practice as students progress through the APPE curriculum, postgraduate training, and into the workforce, where AMS will be employed. Future research should be completed on the impact of required AMS training during APPE rotations, analyzing both student’s understanding and the clinical implications of student participation in a variety of AMS techniques. The impact of required AMS education in didactic and experiential education on future pharmacist involvement with AMS practices should also be analyzed.

## Appendix A. Checklist for Core Elements of Antimicrobial Stewardship—Community/Retail Setting

**Commitment**

Does the facility or organization demonstrate dedication to and accountability for optimizing antibiotic prescribing and patient safety related to antibiotics? ☐Yes ☐No

Select all that apply:

☐Have leadership that focuses on optimizing antibiotic therapy
☐Display public commitments in support of antibiotic stewardship
☐Other ____

**Action**

Pharmacy Practice

What do practitioners at your site evaluate when presented with an antibiotic prescription? (select all that apply)

☐Ask indication if not available
☐Ensure the drug is appropriate for the indication
☐Check for allergic reaction
☐Check dosing is appropriate for infection and other patient characteristics?
☐Check duration of therapy is appropriate?
☐Other ____

What do practitioners at your site do for an allergy with an unknown reaction or poorly documented reaction? (select all that apply)

☐Ask patient what the reaction was?
☐If patient doesn’t remember, does the pharmacist probe for further details (such as when did the reaction occur? Were you hospitalized?)
☐Other ____

What do practitioners at your site counsel on in terms of antibiotic therapy? (select all that apply)

☐Indication
☐Directions on how to take
☐Adverse effects
☐Discuss when improvement should be seen and/or what to do if no improvement?
☐Other

Point of Care Practices

Does your pharmacy site offer vaccinations to patients?  ☐Yes  ☐No

Does your pharmacy site offer any infectious diseases point-of-care tests (such as Group A *Streptococcus* or influenza)?  ☐Yes  ☐No

Which ones? ____

**Safety**

If an adverse reaction is reported to a practitioner at your site, what does the practitioner do with that information? (select all that apply)

☐Report to MedWatch when applicable
☐Document reaction in computer system, if severe
☐Educate patient on how to manage adverse reactions, if non-severe

**Education**

Resources

Do practitioners at your site have access to an electronic health record of an associated health system?  ☐Yes  ☐No

Do practitioners at your site have access to any of the following reference material? (Check all that apply)

☐LexiComp
☐Micromedex
☐Johns Hopkins
☐Sanford Guide
☐UpToDate
☐Other (please indicate):

Provider Education

Do practitioners at your site provide resources to clinicians on evidence-based prescribing?

☐Yes  ☐No

Provide an example _____

Patient Education

Do practitioners at your site provide resources or education to patients about antibiotics (above and beyond the medication insert)?  ☐Yes  ☐No

Provide an example ___ (Example could include educating on virus vs. bacteria, adverse effects, etc.)

Do practitioners at your site provide resources to patients presenting with a cold/viral symptoms?  ☐Yes  ☐No

Check all that apply:
☐Supportive care recommendations
☐Provide education on bacterial vs. viral etiologies

Pharmacy Practitioner Education

Do practitioners participate in continuing education regarding antibiotic therapy on a routine basis?  ☐Yes  ☐No

**Tracking and Reporting**

Does your facility or organization monitor at least one aspect of antibiotic prescribing? (Select all that apply) ☐Yes   ☐No

☐Report percentage of prescriptions that are antibiotics
☐Track interventions with prescriber for antibiotic-related medication problems
☐Set goals surrounding antibiotic assessment (for example, indication known for >90% of prescriptions)

## Appendix B

STUDENT SURVEY

1. Choose your graduating year (dropdown)
  - Class of 2020
  - Class of 2021
  - Class of 2022
2. Which antimicrobial stewardship checklist and reflection did you complete while on IPPE rotations? (Select all that apply)
  - Retail
  - Ambulatory
  - Hospital
3. Which antimicrobial stewardship checklist(s) did you complete prior to learning about antimicrobial stewardship in PHAR 562 (Infectious Diseases 1, Week 10 of Session 3)? (Select all that apply)
  - Retail
  - Ambulatory
  - Hospital
  - I don’t remember
4. Rate your understanding of each antimicrobial stewardship technique/practice before and after completing the IPPE antimicrobial stewardship checklist(s). Rate on a scale of: 1 = Do not understand, 2 = I can explain this antimicrobial stewardship practice, 3 = I can explain and conduct this antimicrobial stewardship practice.
  - Dosing optimization
  - Duration of therapy adjustments
  - Allergy assessments
  - Antibiotic selection recommendations
  - Guideline/policy development
  - Order set implementation
  - IV to PO interchange
  - Antibiotic time-out
  - Computerized alerts
  - Pharmacist education
  - Patient education
  - Prescriber education
  - Assessing and recommending vaccinations
  - Tracking antibiotic use
  - Prospective audit and feedback
  - Antimicrobial restriction
5. How well did the reflection enhance your understanding of implementing antimicrobial stewardship techniques? Rate on a scale of 1 = Did not enhance understanding, 2 = Somewhat enhanced understanding, 3 = Greatly enhanced understanding.
6. How likely are you to implement AMS techniques in your future career? Rate on a scale of 1 = Highly unlikely, 2 = Somewhat unlikely, 3 = Neither unlikely or likely, 4 = Likely, 5 = Highly likely.
7. How often were antimicrobial stewardship techniques observed while you were on rotation?
  - Community Rotation
    i. Every day
    ii. Once per month
    iii. Once per rotation
    iv. Other? Comment
    v. NA
  - Ambulatory Rotation
    vi. Every day
    vii. Once per month
    viii. Once per rotation
    ix. Other? Comment
    x. NA
  - Acute Care Rotation
    xi. Every day
    xii. Once per month
    xiii. Once per rotation
    xiv. Other? Comment
    xv. NA
8. Did you discuss the AMS activity with your preceptor?
  - Community Pharmacy
    xvi. Yes
    xvii. No
  - Ambulatory Care
    xviii. Yes
    xix. No
  - Acute Care
    xx. Yes
    xxi. No
9. What antimicrobial stewardship technique is not currently employed at the site but could be implemented? (Select all that apply; will ask for community, ambulatory, and acute care rotations.) Will have expandable definitions for each (see definitions section).
  - Dosing optimization
  - Duration of therapy adjustments
  - Allergy assessments
  - Antibiotic selection recommendations
  - Guideline/policy development
  - Order set implementation
  - IV to PO interchange
  - Antibiotic time-out (review at 48–72 h)
  - Computerized alerts
  - Pharmacist education
  - Patient education
  - Prescriber education
  - Assessing and recommending vaccinations
  - Tracking antibiotic use
  - Prospective audit and feedback
  - Antimicrobial restriction
10. Comments

**Definitions:**

(a) Introductory pharmacy practice experience (IPPE): A rotation pharmacy students complete during the timeframe of didactic instruction in order to introduce students to the practice of pharmacy in various settings. At MCW School of Pharmacy, students complete seven distinct IPPE rotations. Each IPPE rotation totals a minimum of 80 h and has been intentionally incorporated into the curriculum to occur every Friday for a minimum of 8 h per day for 10 consecutive weeks.
(b) Dosing optimization [7]: Utilizing the pharmacokinetic and pharmacodynamic properties of a drug as well as individual patient characteristics to determine optimal antibiotic dosing. Examples include: renal dose adjustment, dose adjustment for indication, extended infusion beta-lactams, pharmacokinetic dosing of vancomycin/aminoglycosides, etc.
(c) Duration of therapy adjustments [7]: Prolonging or shortening antibiotic duration of therapy based on guideline recommendations and/or patient-specific factors and response to antibiotic therapy.
(d) Allergy assessments [7]: Defining an antibiotic allergy to include reaction type, onset after ingestion/administration, how allergic reaction was treated, how long ago reaction occurred, etc. and documenting information. Could also include conducting penicillin skin testing.
(e) Antibiotic selection recommendations [7]: Initial, de-escalation, or alternative antibiotic therapy recommendations made based on guidelines, patient-specific factors, and/or local susceptibility data
(f) Guideline/policy development [7]: Developing institutional or organizational practices for antibiotic use that take into account national guidelines, patient-specific characteristics, and local susceptibility data.
(g) Order set implementation [7]: An electronic or paper tool that guides institutional or organizational practice for antibiotic use. The order set can assist with antibiotic selection, dosing, route, and duration of therapy.
(h) IV to PO interchange [7]: An assessment of ability for a patient to tolerate enteral antibiotics. If enteral antibiotics can be utilized, intervention to oral therapy from intravenous therapy should be made.
(i) Antibiotic time-out [7]: A routine, structured review of antimicrobial therapy at a set time (usually 48–72 h after initiation of empiric antimicrobial therapy).
(j) Computerized alerts [7]: Notifications of potential interactions or misuse of antibiotics that are displayed to prescribers at the time of antibiotic ordering.
(k) Pharmacist education [7]: Educating pharmacists on antimicrobial stewardship techniques, resistance, and antimicrobial best practices. Education could be either active (e.g., direct, real-time feedback) or passive (e.g., email).
(l) Patient education [7]: Educating patients on the appropriate use of antimicrobials, antimicrobial resistance, the importance of antimicrobial stewardship, the difference between viral and bacterial infections, etc. Education could be either active (e.g., direct, real-time feedback) or passive (e.g., email).
(m) Prescriber education [7]: Educating prescribers on the appropriate use of antimicrobials, antimicrobial resistance, the importance of antimicrobial stewardship, the difference between viral and bacterial infections, etc. Education could be either active (e.g., direct, real-time feedback or as prospective audit and feedback) or passive (e.g., email).
(n) Assessing and recommending vaccinations: Analyzing patient data to determine vaccine eligibility and recommending immunizations for patients. Could involve vaccine administration.
(o) Tracking antibiotic use [7]: Any system in place that allows for antibiotic use or prescriptions to be quantified.
(p) Prospective audit and feedback [7]: Structured review of antibiotic utilization, often conducted after antibiotics have already been prescribed and possibly administered.
(q) Antimicrobial restriction [7]: Limiting use of antimicrobials for specific indications or requiring approval by an infectious diseases practitioner before prescription of that antimicrobial can occur.

**PRECEPTOR SURVEY**

1. Select your practice setting
  - Community/retail pharmacy
  - Ambulatory clinic
  - Acute care
  - Other (Please specify)
2. How long have you been a practicing pharmacist?
  - 0–5 years
  - 6–10 years
  - 11–20 years
  - >20 years
3. Did you receive didactic education on antimicrobial stewardship in pharmacy school?
  - Yes
    - If Yes—how many hours?
    - If Yes—please describe.
  - No
4. How many continuing education sessions for antimicrobial stewardship have you attended?
  - None
  - 0–5
  - 6–10
  - >10
5. Did you discuss the antimicrobial stewardship assignment with any of your MCW School of Pharmacy IPPE students?
  - Yes
    - If yes, rate your understanding of the following antimicrobial stewardship practices before and after the IPPE rotation. Rate on a scale of: 1 = Do not understand, 2 = I can explain this antimicrobial stewardship practice, 3 = I can explain and conduct this antimicrobial stewardship practice.
      1. Dosing optimization
      2. Duration of therapy adjustments
      3. Allergy assessments
      4. Antibiotic selection recommendations
      5. Guideline/policy development
      6. Order set implementation
      7. IV to PO interchange
      8. Antibiotic time-out
      9. Computerized alerts
      10. Pharmacist education of antimicrobial stewardship principles and practices
      11. Patient education of antimicrobial stewardship principles
      12. Prescriber education of antimicrobial stewardship principles and practices
      13. Assessing and recommending vaccinations
      14. Tracking antibiotic use
      15. Prospective audit and feedback
      16. Antimicrobial restriction
  - No
    - If No, would you be interested in utilizing a checklist to assess current antimicrobial stewardship techniques at your practice site?
      1. Yes—if yes, please contact pharmacyee@mcw.edu
      2. No
    - If No, would you be interested in working with an IPPE student on this activity?
      1. Yes
      2. No
6. What antimicrobial stewardship techniques does your site routinely utilize? (Select all that apply)—Will have expandable definitions for each (see definitions section).
  - Dosing optimization
  - Duration of therapy adjustments
  - Allergy assessments
  - Antibiotic selection recommendations
  - Guideline/policy development
  - Order set implementation
  - IV to PO interchange
  - Antibiotic time-out (review at 48–72 h)
  - Computerized alerts
  - Pharmacist education
  - Patient education
  - Prescriber education
  - Assessing and recommending vaccinations
  - Tracking antibiotic use
  - Prospective audit and feedback
  - Antimicrobial restriction
7. What antimicrobial stewardship technique are you most likely to implement that you are not currently utilizing in your current practice? Select only one option. Will have expandable definitions for each (see definitions section).
  - Dosing optimization
  - Duration of therapy adjustments
  - Allergy assessments
  - Antibiotic selection recommendations
  - Guideline/policy development
  - Order set implementation
  - IV to PO interchange
  - Antibiotic time-out (review at 48–72 h)
  - Computerized alerts
  - Pharmacist education
  - Patient education
  - Prescriber education
  - Assessing and recommending vaccinations
  - Tracking antibiotic use
  - Prospective audit and feedback
  - Antimicrobial restriction
8. Comments (Free Text)

**Definitions:**

(a) Introductory pharmacy practice experience (IPPE): A rotation pharmacy students complete during the timeframe of didactic instruction in order to introduce students to the practice of pharmacy in various settings. At MCW School of Pharmacy, students complete seven distinct IPPE rotations. Each IPPE rotation totals a minimum of 80 h and has been intentionally incorporated into the curriculum to occur every Friday for a minimum of 8 h per day for 10 consecutive weeks.
(b) Dosing optimization [7]: Utilizing the pharmacokinetic and pharmacodynamic properties of a drug as well as individual patient characteristics to determine optimal antibiotic dosing. Examples include: renal dose adjustment, dose adjustment for indication, extended infusion beta-lactams, pharmacokinetic dosing of vancomycin/aminoglycosides, etc.
(c) Duration of therapy adjustments [7]: Prolonging or shortening antibiotic duration of therapy based on guideline recommendations and/or patient-specific factors and response to antibiotic therapy.
(d) Allergy assessments [7]: Defining an antibiotic allergy to include reaction type, onset after ingestion/administration, how allergic reaction was treated, how long ago reaction occurred, etc. and documenting information. Could also include conducting penicillin skin testing.
(e) Antibiotic selection recommendations [7]: Initial, de-escalation, or alternative antibiotic therapy recommendations made based on guidelines, patient-specific factors, and/or local susceptibility data
(f) Guideline/policy development [7]: Developing institutional or organizational practices for antibiotic use that take into account national guidelines, patient-specific characteristics, and local susceptibility data.
(g) Order set implementation [7]: An electronic or paper tool that guides institutional or organizational practice for antibiotic use. The order set can assist with antibiotic selection, dosing, route, and duration of therapy.
(h) IV to PO interchange [7]: An assessment of ability for a patient to tolerate enteral antibiotics. If enteral antibiotics can be utilized, intervention to oral therapy from intravenous therapy should be made.
(i) Antibiotic time-out [7]: A routine, structured review of antimicrobial therapy at a set time (usually 48–72 h after initiation of empiric antimicrobial therapy).
(j) Computerized alerts [7]: Notifications of potential interactions or misuse of antibiotics that are displayed to prescribers at the time of antibiotic ordering.
(k) Pharmacist education [7]: Educating pharmacists on antimicrobial stewardship techniques, resistance, and antimicrobial best practices. Education could be either active (e.g., direct, real-time feedback) or passive (e.g., email).
(l) Patient education [7]: Educating patients on the appropriate use of antimicrobials, antimicrobial resistance, the importance of antimicrobial stewardship, the difference between viral and bacterial infections, etc. Education could be either active (e.g., direct, real-time feedback) or passive (e.g., email).
(m) Prescriber education [7]: Educating prescribers on the appropriate use of antimicrobials, antimicrobial resistance, the importance of antimicrobial stewardship, the difference between viral and bacterial infections, etc. Education could be either active (e.g., direct, real-time feedback or as prospective audit and feedback) or passive (e.g., email).
(n) Assessing and recommending vaccinations: Analyzing patient data to determine vaccine eligibility and recommending immunizations for patients. Could involve vaccine administration.
(o) Tracking antibiotic use [7]: Any system in place that allows for antibiotic use or prescriptions to be quantified.
(p) Prospective audit and feedback [7]: Structured review of antibiotic utilization, often conducted after antibiotics have already been prescribed and possibly administered.
(q) Antimicrobial restriction [7]: Limiting use of antimicrobials for specific indications, or requiring approval by an infectious diseases practitioner before prescription of that antimicrobial can occur.
